# Long-term safety and effectiveness of romiplostim in patients with refractory aplastic anemia: a nationwide, all-case, post-marketing surveillance study in Japan

**DOI:** 10.1007/s00277-026-07062-5

**Published:** 2026-05-30

**Authors:** Mitsuhiro Itagaki, Hiroshi Kuwazawa, Shun Sasaki, Moe Matsuura, Yudai Tanaka, Naoshi Obara

**Affiliations:** 1 Department of Hematology, Hiroshima Red Cross Hospital & Atomic-bomb Survivors Hospital, Hiroshima, Japan; 2 Kyowa Kirin Co., Ltd., Tokyo, Japan; 3 Department of Medical Sciences, Laboratory Hematology, Institute of Medicine, University of Tsukuba, Ibaraki, Japan

**Keywords:** Thrombopoietin receptor agonist, Romiplostim, Aplastic anemia, Hematological response, Post-marketing surveillance study

## Abstract

This all-case post-marketing surveillance study in Japan evaluated the safety and effectiveness of romiplostim, a thrombopoietin receptor agonist, in patients with aplastic anemia (AA), a rare disease characterized by pancytopenia. All patients with refractory AA who initiated romiplostim from June 2019 to September 2021 were registered. Romiplostim was administered subcutaneously once weekly for 52 weeks. Observation lasted 52 weeks or until 4 weeks after discontinuation. Occurrences of bone marrow fibrosis and hematopoietic tumors were monitored for up to 2 years after romiplostim initiation. Data from 470 patients at 220 institutions were analyzed. The mean age ± standard deviation was 66.27 ± 16.89 years, and 43.40% were male. Romiplostim was discontinued in 219/470 patients (46.60%). Adverse events (AEs) occurred in 57.02% of patients, and adverse drug reactions (ADRs) occurred in 18.09%. AEs of special interest classified as ADRs included thromboembolism (1.49%), hematopoietic tumor (1.28%), and hemorrhage (0.46%). During follow-up, hematopoietic tumors as ADRs were observed in 2/310 patients (0.65%). Hematological response was achieved in 257/327 patients (78.59%) at 27 weeks and 205/244 patients (84.02%) at 52 weeks. Platelet transfusion independence or reduced requirement was achieved in 164/213 patients (77.00%) at 27 weeks and 133/160 patients (83.13%) at 52 weeks, while red blood cell transfusion independence or reduced requirement was achieved in 194/247 patients (78.54%) and 162/186 patients (87.10%), respectively. Long-term romiplostim treatment was safe and effective in patients with refractory AA in a real-world setting.

Registration: University Hospital Medical Information Network (UMIN000056465), 16 December, 2024.

**Graphical Abstract:**

**Figure:**
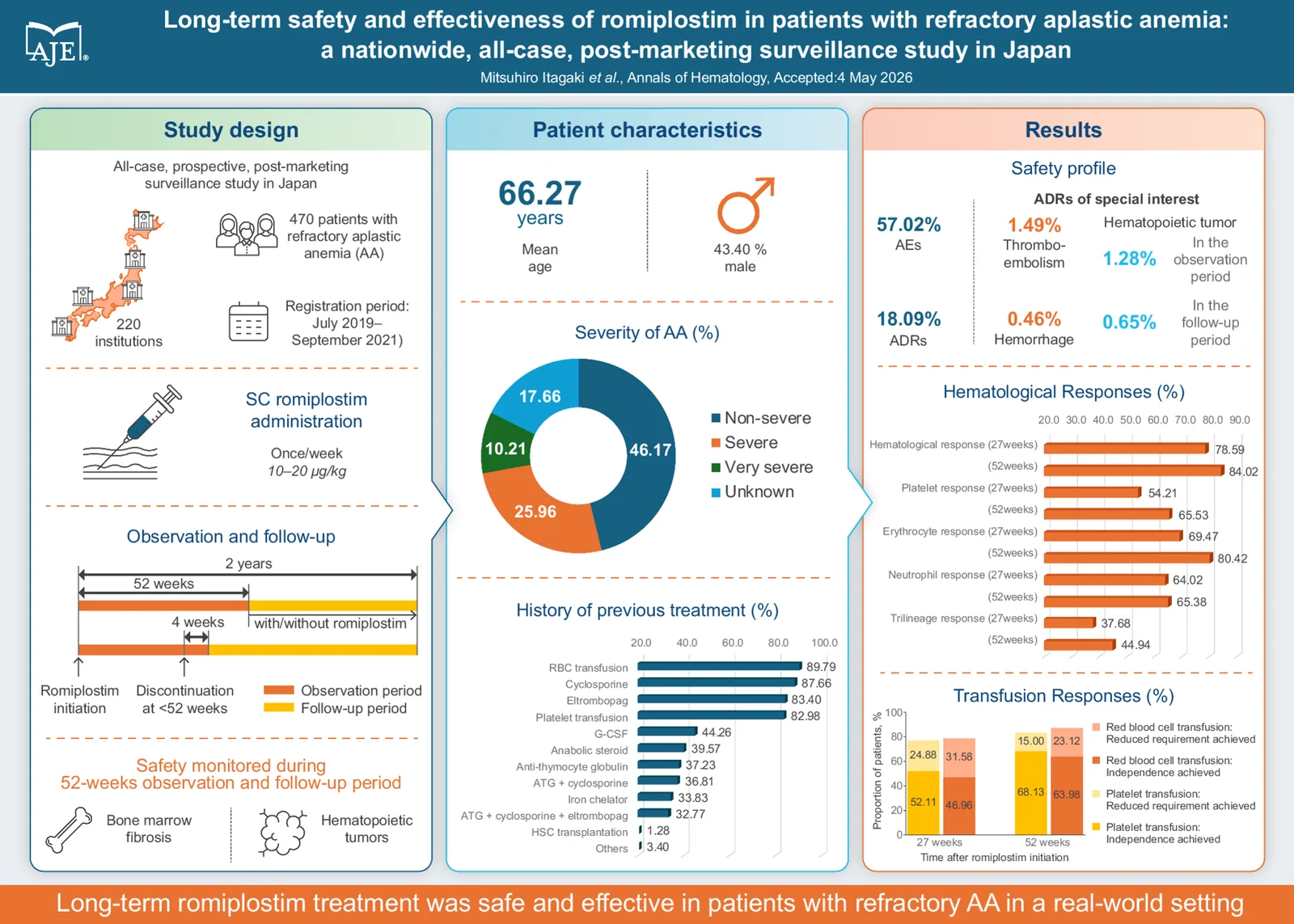

## Introduction

Aplastic anemia (AA) is a rare but severe hematological disorder characterized by pancytopenia and bone marrow hypoplasia. The incidence of AA is 2- to 3-fold higher in Asian countries (approximately 5/million person-years) than in Western countries (approximately 2/million person-years) [1, 2]. AA may be congenital (e.g., Fanconi anemia) or acquired; most acquired cases are idiopathic and considered autoimmune in nature. Treatment options include hematopoietic stem cell transplantation, immunosuppressive therapy (IST) with cyclosporin A (CsA) and anti-thymocyte globulin (ATG), blood transfusion, granulocyte colony-stimulating factor (G-CSF), and thrombopoietin receptor agonists (TPO-RAs). However, hematopoietic stem cell transplantation is not suitable for all patients and is associated with a transplantation-related mortality rate of 10%–20% [3, 4]. The response rate to IST is 33%–57%, and among long-term survivors, 5%–10% develop hematopoietic malignancies such as myelodysplastic syndromes (MDS) or acute myeloid leukemia (AML), while 10%–15% develop paroxysmal nocturnal hemoglobinuria [4–7]. Blood transfusion is supportive rather than curative and is associated with risks of infection, refractoriness because of undesired antibody development, and secondary hemochromatosis due to chronic transfusion [8–10]. The addition of G-CSF to immunosuppressants can transiently increase the neutrophil count and reduce infection events, but does not improve responses to immunosuppressants or event-free, relapse-free, or overall survival [11, 12].

Treatment remains challenging for patients with AA refractory to conventional therapy (refractory AA) or for whom IST is not applicable because of long disease duration. TPO-RAs, such as eltrombopag or romiplostim, have been developed to treat such patients. Activation of the TPO receptor, cellular myeloproliferative leukemia (c-Mpl), stimulates megakaryopoiesis and increases platelet production [13, 14]. Eltrombopag and romiplostim demonstrated thrombopoietic efficacy in patients with chronic immune thrombocytopenia (ITP) [15–17] and were approved for this indication. Beyond its effects on megakaryocyte progenitors, c-Mpl signaling regulates early hematopoiesis by stimulating hematopoietic stem cells, inducing erythroid and neutrophil production in addition to platelets [18–22]. Accordingly, TPO-RAs have emerged as promising agents for the treatment of pancytopenia in AA, and both eltrombopag [23–25] and romiplostim [16, 17] have shown efficacy in restoring trilineage blood cells in clinical studies and have been approved for AA.

Romiplostim has been used to treat ITP since its first approval in Australia in 2008 (January 2011 in Japan). In Japan, indications were subsequently expanded to refractory AA (June 2019) and treatment-naïve AA [26] (September 2023), with later expansion to other countries. Romiplostim is a recombinant protein fused to human IgG1-Fc and has no sequence homology to endogenous TPO [27]. Because more than half of IST-refractory patients did not respond to eltrombopag in clinical studies [23, 24], romiplostim is expected to be an alternative effective therapy [28–30], also for patients with IST- or eltrombopag-refractory AA.

This post-marketing surveillance study was conducted to collect real-world safety and effectiveness data on romiplostim in patients with refractory AA who were not included in clinical studies and were treated with romiplostim after its approval for this indication in Japan.

## Methods

### Study design and patients

This study was a post-marketing surveillance conducted as an all-case, non-interventional, prospective, real-world observational study in Japan. It was registered with the University Hospital Medical Information Network (UMIN000056465).

All patients with refractory AA who received romiplostim during the registration period were centrally registered. The registration period was from July 2019 (approval of romiplostim for AA) until September 2021. For the safety and effectiveness analyses, the following cases were excluded from the fixed dataset: patients whose primary disease was not AA, those who did not receive romiplostim, participants in clinical trials involving romiplostim, and patients treated at institutions that did not provide consent for data publication.

**Fig. 1:**
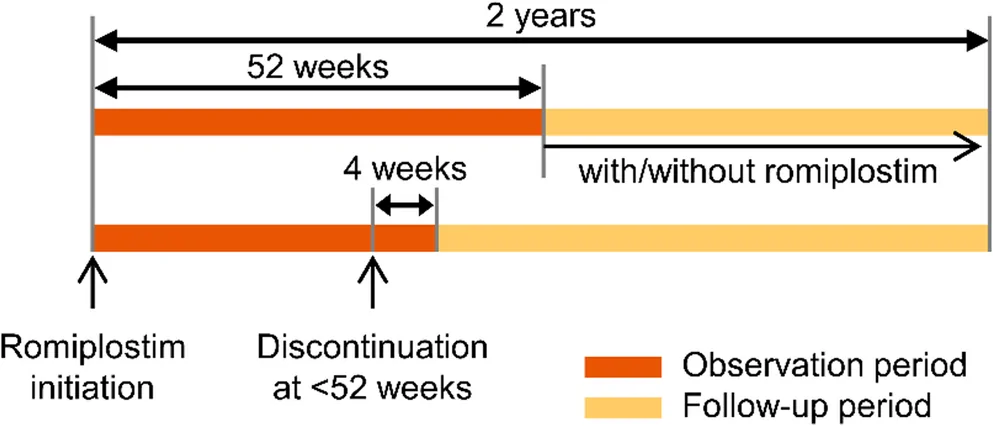
Study design. Observation and follow-up periods for patients who completed 52-week treatment (upper bar) and who discontinued treatment before 52 weeks (lower bar)

The observation period was defined as 52 weeks after initiation of romiplostim, or 4 weeks after discontinuation, if applicable. The follow-up period was defined as the period starting from the end of the observation period until 2 years after romiplostim initiation (Fig. 1). Following baseline data were collected: the patients’ demographic characteristics, AA severity, laboratory test values, and volumes of red blood cell (RBC) or platelet transfusions during the 8 weeks before treatment initiation. During the observation period, data were collected on romiplostim treatment, safety, laboratory test values, and RBC or platelet transfusion volumes. During the follow-up period, the occurrence of two adverse events (AEs) of special interest (bone marrow fibrosis and hematopoietic tumor) was recorded.

### Safety

Information was collected on AEs, coded according to MedDRA/J version 27.0, along with their dates of occurrence, severity, causal relationship to romiplostim, and outcomes. Severity and causal relationships were assessed by study physicians at each site.

Four AEs were predefined as AEs of special interest: hemorrhage, thromboembolism, bone marrow fibrosis, and hematopoietic tumor. Among these, occurrences of bone marrow fibrosis and hematopoietic tumor were additionally monitored during the follow-up period.

### Effectiveness

Based on laboratory test values (including erythrocyte count, hemoglobin concentration, leukocyte count, platelet count, differential leukocyte count, reticulocyte count), responses to romiplostim in each blood cell lineage (platelets, erythrocytes, and neutrophils), as well as hematological and trilineage responses, were assessed. From platelet and RBC transfusion data, achievement of transfusion independence or reduced transfusion requirements was determined. Definitions of platelet, erythrocyte, and neutrophil responses are summarized in Table 1.

**Table 1:** Definitions for achievement of hematological and transfusion responses to romiplostim

Hematological responses
Platelet response
• Platelet count increase of ≥ 20,000/µL from baseline, or • Platelet count increase of ≥ 10,000/µL and ≥ 100% from baseline, or • Platelet transfusion independence for ≥ 8 consecutive weeks
Erythrocyte response
• Hemoglobin concentration increase of ≥ 1.5 g/dL from a baseline value of < 9 g/dL, or • Decrease of ≥ 800 mL in the cumulative volume of RBC transfusion over 8 consecutive weeks, compared with the total volume during the 8 consecutive weeks before baseline, in patients who received RBC transfusions before initiation of romiplostim treatment
Neutrophil response
• Neutrophil count increase of 100% from baseline value of < 500/µL, or • Neutrophil count increase of ≥ 500/µL from baseline value of < 1,000/µL
Hematological response
• Achievement of any of the three blood cell responses (platelet, erythrocyte, and/or neutrophil)
Trilineage response
• Achievement of all three blood cell responses (platelet, erythrocyte, and neutrophil)
Transfusion responses
Transfusion independence (erythrocyte/platelet) in patients who were transfusion-dependent 8 weeks before romiplostim initiation
• No requirement for transfusion for 8 consecutive weeks
Reduced transfusion requirement (erythrocyte/platelet) in patients who were transfusion-dependent during the 8 weeks before romiplostim initiation
• Decrease in transfusion volume over 8 consecutive weeks

### Romiplostim administration

Romiplostim (Romiplate^®^; Kyowa Kirin Co., Ltd., Tokyo, Japan) was administered subcutaneously once weekly. The initial dose was 10 μg/kg, and subsequent doses were adjusted according to patients’ hematological status, up to a maximum dose of 20 μg/kg, as specified in the package insert [31]. Dates of administration, dose adjustments, discontinuation and reasons (if applicable), and availability of follow-up were recorded during the observation period.

### Statistics

Patient demographic characteristics, safety, and effectiveness were descriptively summarized. AA severity at baseline was evaluated using laboratory test values according to the modified Camitta criteria [32]. Time from romiplostim initiation to achievement of hematological, platelet, erythrocyte, neutrophil responses, and independence or reduced requirements of RBC or platelet transfusion was estimated using the Kaplan-Meier method, and 95% confidence intervals (CIs) were calculated.

## Results

### Patients

**Fig. 2:**
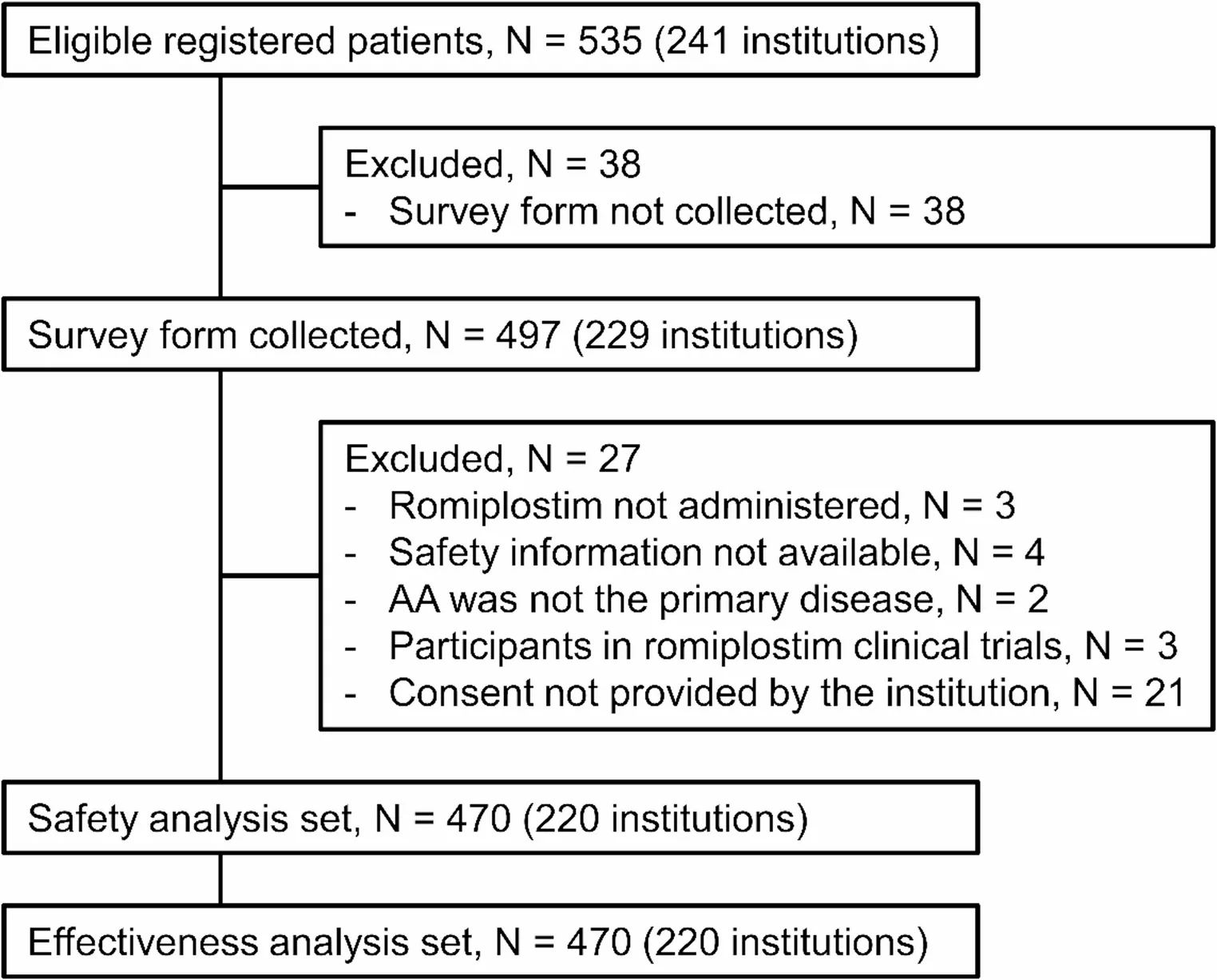
Patients’ disposition

Survey forms were collected from 497 eligible registered patients at 229 institutions. After application of the exclusion criteria, 470 patients at 220 institutions were included in the safety and effectiveness analyses (Fig. 2). The patients’ demographic characteristics are summarized in Table 2. Of the 470 patients analyzed, 204 (43.40%) were male and 303 (64.47%) were aged ≥ 65 years. Their mean age ± standard deviation (SD) was 66.27 ± 16.89 years. According to the modified Camitta criteria, AA was non-severe in 217/470 patients (46.17%), severe in 122/470 patients (25.96%), and very severe in 48/470 patients (10.21%). Most patients had previously received IST with cyclosporine (412/470 patients, 87.66%) and TPO-RA therapy with eltrombopag (392/470 patients, 83.40%).

**Table 2:** Demographic characteristics of patients at baseline

Characteristics	
Total, n (%)	470 (100.00)
Sex, n (%)	
Male	204 (43.40)
Female	266 (56.60)
Age, years	
n	469
Mean ± SD	66.27 ± 16.89
Median	71.00
Age categories, years	
< 20	9 (1.91)
≥ 20 and < 40	33 (7.02)
≥ 40 and < 65	124 (26.38)
≥ 65	303 (64.47)
Unknown	1 (0.21)
Time since diagnosis, years	
n	394
Mean ± SD	2.48 ± 3.76
Median	1.08
Severity of AA^a^, n (%)	
Non-severe	217 (46.17)
Severe	122 (25.96)
Very severe	48 (10.21)
Unknown	83 (17.66)
History of previous treatment, n (%)	
RBC transfusion	422 (89.79)
Cyclosporine	412 (87.66)
Eltrombopag	392 (83.40)
Platelet transfusion	390 (82.98)
G-CSF	208 (44.26)
Anabolic steroid	186 (39.57)
Anti-thymocyte globulin	175 (37.23)
ATG + cyclosporine	173 (36.81)
Iron chelator	159 (33.83)
ATG + cyclosporine + eltrombopag	154 (32.77)
Others	16 (3.40)
Hematopoietic stem cell transplantation	6 (1.28)
Reticulocyte count, ×10^4^/µL	
n	391
Mean ± SD	5.20 ± 31.95
Median	2.04
Platelet count, ×10^4^/µL	
n	466
Mean ± SD	2.30 ± 4.14
Median	1.30
Hemoglobin concentration, g/dL	
n	465
Mean ± SD	7.87 ± 1.59
Median	7.60
Neutrophil count, /µL	
n	453
Mean ± SD	1211.96 ± 1324.93
Median	911.24

^a^Severity was assessed using the modified Camitta criteria [32]

*AA* aplastic anemia, *ATG* anti-thymocyte globulin, *G-CSF* granulocyte colony-stimulating factor, *RBC* red blood cell, *SD* standard deviation

### Romiplostim administration

**Fig. 3:**
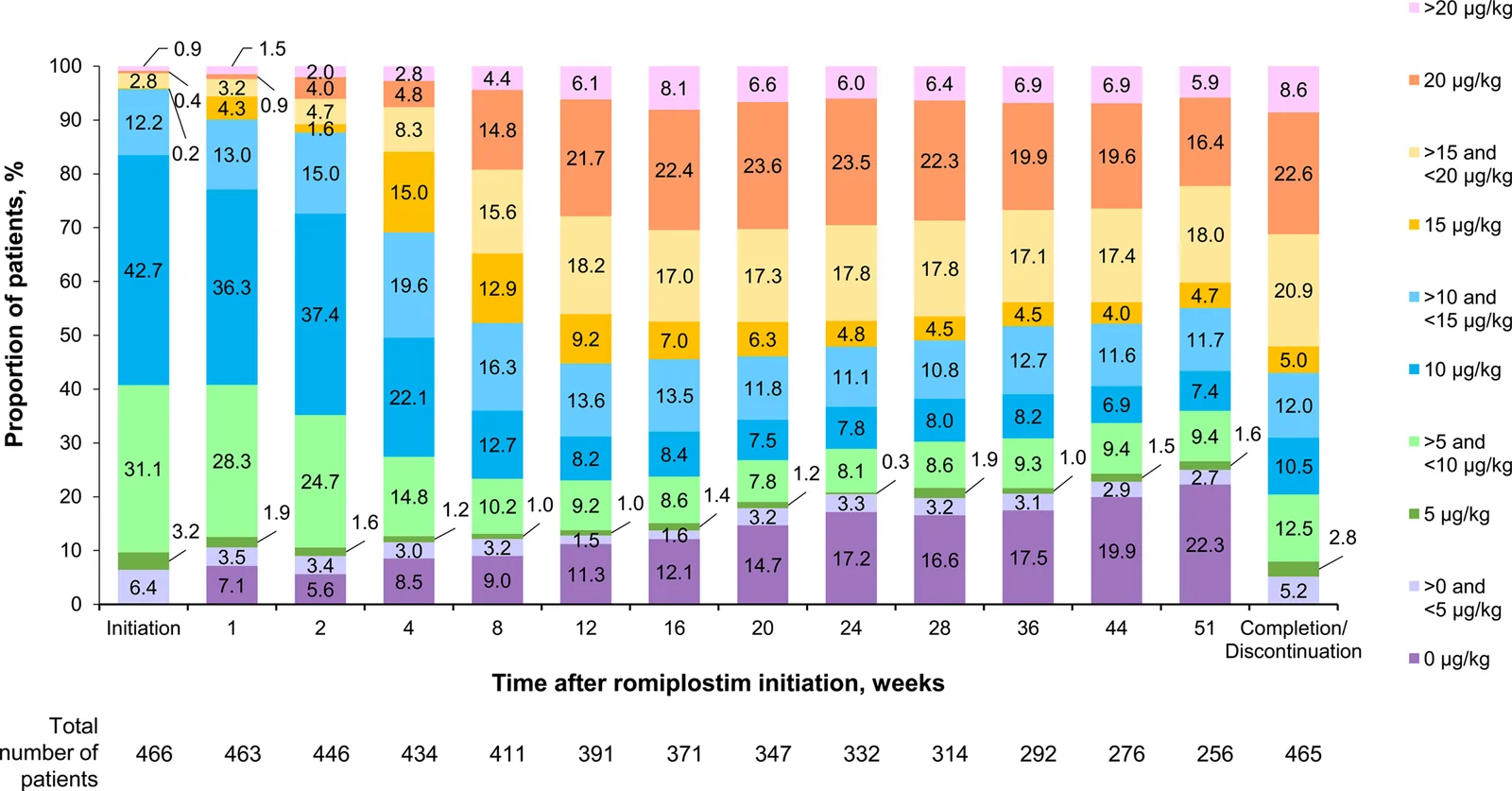
Time-course changes in romiplostim dose

At treatment initiation, the mean romiplostim dose ± SD was 9.44 ± 3.00 µg/kg, and the mean treatment duration was 256.69 ± 132.03 days. Approximately 75% of patients received an initial dose of > 5 µg/kg and ≤ 10 µg/kg. Thereafter, the proportion of patients receiving > 15 µg/kg gradually increased, and at treatment completion or discontinuation, 242/465 patients (52.04%) had received > 15 µg/kg. Time-course changes in the weekly dose distribution are presented in Fig. 3.

Romiplostim was discontinued in 219/470 patients (46.60%), with a mean time to discontinuation of 145.95 ± 107.20 days. The most common reason was lack of effectiveness (89/470 patients, 18.94%), followed by AEs (61/470 patients, 12.98%) (Table 3). Among AEs leading to discontinuation, adverse drug reactions (ADRs) occurred in 18/470 patients (3.83%), including liver dysfunction in 3/470 patients (0.64%).

**Table 3:** Discontinuation of romiplostim treatment

Total	*n* (%)	
	470 (100.00)	
Discontinued	219 (46.60)	
Reasons for treatment discontinuation		
Lack of effectiveness	89 (18.94)	
Highest dose in discontinued cases during treatment, µg/kg		
≤ 5	1/89 (1.12)	
> 5 and ≤ 10	10/89 (11.24)	
> 10 and ≤ 15	12/89 (13.48)	
> 15 and ≤ 20	56/89 (62.92)	
> 20	10/89 (11.24)	
Adverse events	61 (12.98)	
Adverse drug reactions	18 (3.83)	
Improved symptoms	20 (4.26)	
Patient’s request	10 (2.13)	
Hospital transfer	23 (4.89)	
Others	36 (7.66)	

### Safety

AEs and ADRs occurring during the observation and follow-up periods are summarized in Table 4. AEs occurred in 268/470 patients (57.02%) and 160/470 patients (34.04%) experienced serious AEs. During the observation period, AEs of special interest included hemorrhage (13/219 patients, 5.94%), thromboembolism (11/470 patients, 2.34%), and hematopoietic tumor (11/470 patients, 2.34%). During follow-up period, 310 patients were followed and hematopoietic tumor was observed in 5/310 patients (1.61%). No patient developed bone marrow fibrosis throughout the study period.

**Table 4:** Adverse events and adverse drug reactions during observation and follow-up periods

	Observation period		Follow-up period^a^	
	Number of patients, *n* (%)	Number of events, *n*	Number of patients, *n* (%)	Number of events, *n*
Total	470	–	310	–
Discontinued	219	–	–	–
Adverse events	268 (57.02)	696	5 (1.61)	5
Serious adverse events	160 (34.04)	282	4 (1.29)	4
Adverse events resulting in death	63 (13.40)	79	0 (0.00)	0
Adverse events of special interest	34 (7.23)	36	5 (1.61)	5
Hemorrhage^b^	13 (5.94)^c^	14	–	–
Thromboembolism	11 (2.34)	12	–	–
Bone marrow fibrosis	0 (0.00)	0	0 (0.00)	0
Hematopoietic tumor	11 (2.34)	11	5 (1.61)	5
Adverse drug reactions	85 (18.09)	150	2 (0.65)	2
Serious adverse drug reactions	25 (5.32)	33	2 (0.65)	2
Adverse drug reactions resulting in death	8 (1.70)	10	0 (0.00)	0
Adverse drug reactions of special interest	14 (2.98)	14	2 (0.65)	2
Hemorrhage^b^	1 (0.46)^c^	1	–	–
Thromboembolism	7 (1.49)	7	–	–
Bone marrow fibrosis	0 (0.00)	0	0 (0.00)	0
Hematopoietic tumor	6 (1.28)	6	2 (0.65)	2

^a^During the follow-up period, information was collected only on bone marrow fibrosis and hematopoietic tumors

^b^Hemorrhage events observed after romiplostim discontinuation

^c^Proportion of patients who experienced hemorrhage among the 219 patients who discontinued treatment

ADRs were reported in 85/470 patients (18.09%), with the most frequent events listed in Table 5. Among AEs of special interest, events classified as ADRs occurred in 14/470 patients (2.98%) during the observation period, including hemorrhage (1/219 patients, 0.46%), thromboembolism (7/470 patients, 1.49%; all thrombocytosis-related), and hematopoietic tumor (6/470 patients, 1.28%). Liver disorder as an ADR was uncommon (11/470 patients, 2.34%). During follow-up period, hematopoietic tumor was reported as an ADR in 2/310 patients (0.65%).

**Table 5:** Adverse drug reactions observed in ≥ 4 patients

Total number of patients	*n* (%)
	470
Adverse drug reactions	85 (18.09)
Platelet count increased	6 (1.28)
Headache	5 (1.06)
Aplastic anemia	4 (0.85)
Nausea	4 (0.85)
Liver disorder	4 (0.85)
Myalgia	4 (0.85)
Renal impairment	4 (0.85)

### Effectiveness

**Fig. 4:**
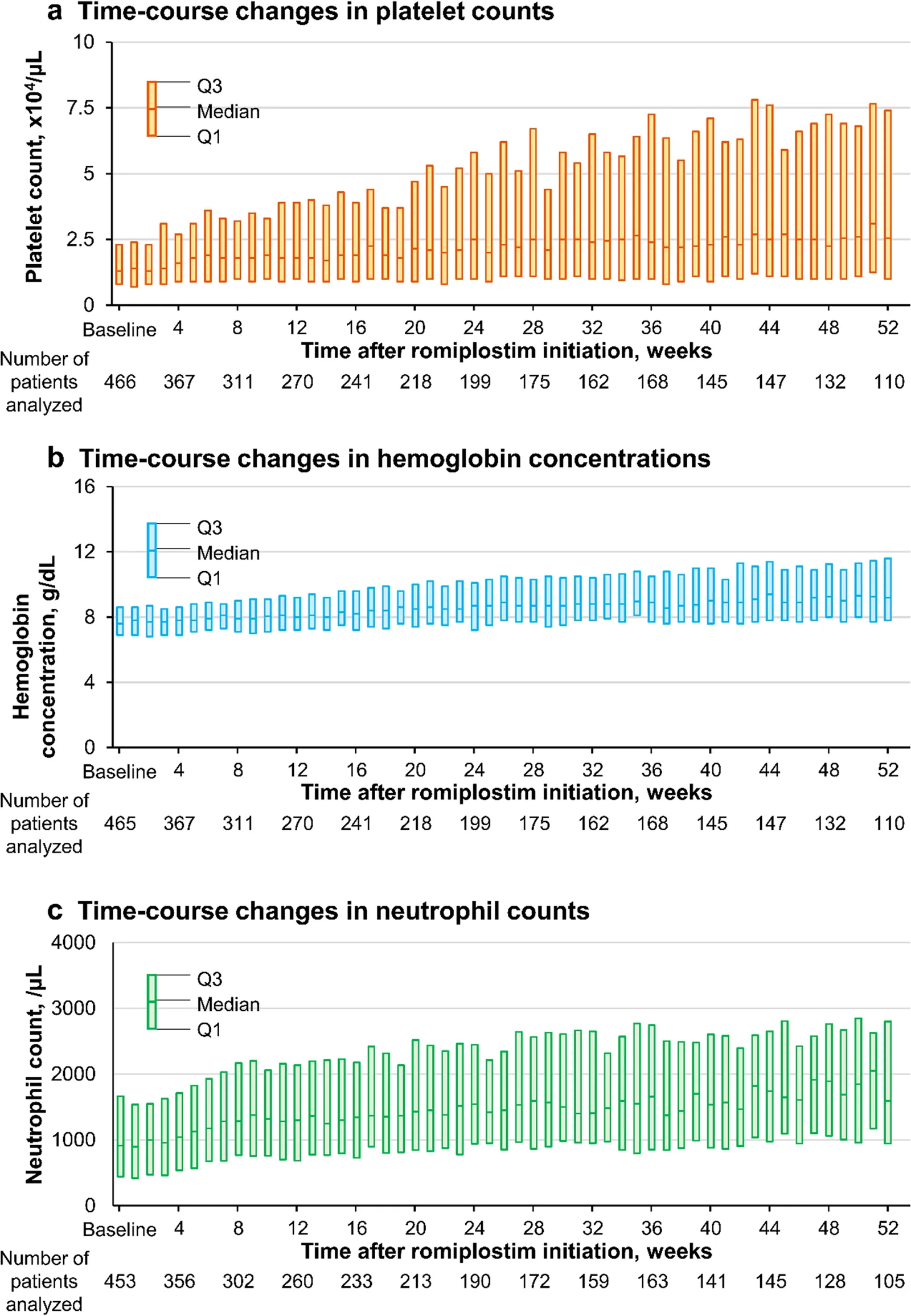
Time-course changes in trilineage blood cells. Time-course changes in (**a**) platelet count, (**b**) hemoglobin concentration, and (**c**) neutrophil count

Time-course changes in the platelet count, hemoglobin concentration, and neutrophil count are presented in Fig. 4. At baseline, the median values were 1.30 × 10^4^/µL for the platelet count, 7.60 g/dL for the hemoglobin concentration, and 911.24/µL for the neutrophil count (Table 2). These values increased gradually to 52 weeks after initiation of romiplostim treatment. The median values at 52 weeks were 2.55 × 10^4^/µL for the platelet count, 9.20 g/dL for the hemoglobin concentration, and 1595.00/µL for the neutrophil count.

A hematological response was achieved in 257/327 patients (78.59%) at 27 weeks and in 205/244 patients (84.02%) at 52 weeks. A trilineage response was achieved in 52/138 patients (37.68%) at 27 weeks and in 40/89 patients (44.94%) at 52 weeks (Table 6). Hematological responses categorized by baseline AA severity and AA treatment history are summarized in Tables 7 and 8, respectively. At 52 weeks, hematological responses were achieved in 110/130 patients (84.62%) with non-severe AA, 47/60 patients (78.33%) with severe AA, and 11/13 patients (84.62%) with very severe AA, indicating consistent responses across severity categories (Table 7). Response rates were also comparable across treatment histories, including patients with and without prior eltrombopag exposure (Table 8).

**Table 6:** Hematological responses

	27 weeks	52 weeks
Hematological response^a^		
Analyzed, n	327	244
Achieved, n (%)	257 (78.59)	205 (84.02)
Platelet response		
Analyzed, n	321	235
Achieved, n (%)	174 (54.21)	154 (65.53)
Erythrocyte response		
Analyzed, n	262	189
Achieved, n (%)	182 (69.47)	152 (80.42)
Neutrophil response		
Analyzed, n	164	104
Achieved, n (%)	105 (64.02)	68 (65.38)
Trilineage response^b^		
Analyzed, n	138	89
Achieved, n (%)	52 (37.68)	40 (44.94)

^a^Achievement of any of the three blood cell responses (platelet, erythrocyte, and/or neutrophil)

^b^Achievement of all three blood cell responses (platelet, erythrocyte, and neutrophil)

**Table 7:** Hematological, trilineage, and individual blood cell responses by AA severity

	Non-severe	Severe	Very severe
**27 weeks**			
Hematological response			
Analyzed, n	168	84	22
Achieved, n (%)	133 (79.17)	63 (75.00)	20 (90.91)
Platelet response			
Analyzed, n	167	82	19
Achieved, n (%)	99 (59.28)	36 (43.90)	7 (36.84)
Erythrocyte response			
Analyzed, n	119	81	18
Achieved, n (%)	88 (73.95)	50 (61.73)	11 (61.11)
Neutrophil response			
Analyzed, n	58	62	20
Achieved, n (%)	39 (67.24)	36 (58.06)	16 (80.00)
Trilineage response			
Analyzed, n	46	58	15
Achieved, n (%)	21 (45.65)	18 (31.03)	6 (40.00)
**52 weeks**			
Hematological response			
Analyzed, n	130	60	13
Achieved, n (%)	110 (84.62)	47 (78.33)	11 (84.62)
Platelet response			
Analyzed, n	127	56	13
Achieved, n (%)	90 (70.87)	31 (55.36)	6 (46.15)
Erythrocyte response			
Analyzed, n	90	54	12
Achieved, n (%)	77 (85.56)	41 (75.93)	6 (50.00)
Neutrophil response			
Analyzed, n	37	37	13
Achieved, n (%)	24 (64.86)	23 (62.16)	11 (84.62)
Trilineage response			
Analyzed, n	30	34	12
Achieved, n (%)	15 (50.00)	15 (44.12)	5 (41.67)

*AA* aplastic anemia

**Table 8:** Hematological, trilineage, and individual blood cell responses by AA treatment history

	ATG	Cyclosporine	Eltrombopag	ATG + Cyclosporine	ATG + Cyclosporine + Eltrombopag
**27 weeks**					
Hematological response					
Analyzed, n	128	289	273	127	111
Achieved, n (%)	102 (79.69)	224 (77.51)	211 (77.29)	101 (79.53)	88 (79.28)
Platelet response					
Analyzed, n	124	283	268	123	107
Achieved, n (%)	74 (59.68)	150 (53.00)	139 (51.87)	73 (59.35)	62 (57.94)
Erythrocyte response					
Analyzed, n	101	233	220	101	91
Achieved, n (%)	73 (72.28)	163 (69.96)	154 (70.00)	73 (72.28)	66 (72.53)
Neutrophil response					
Analyzed, n	72	145	132	71	63
Achieved, n (%)	48 (66.67)	93 (64.14)	86 (65.15)	47 (66.20)	42 (66.67)
Trilineage response					
Analyzed, n	59	125	111	59	52
Achieved, n (%)	25 (42.37)	49 (39.20)	41 (36.94)	25 (42.37)	22 (42.31)
**52 weeks**					
Hematological response					
Analyzed, n	90	215	200	90	79
Achieved, n (%)	75 (83.33)	176 (81.86)	163 (81.50)	75 (83.33)	65 (82.28)
Platelet response					
Analyzed, n	86	207	193	86	75
Achieved, n (%)	58 (67.44)	133 (64.25)	125 (64.77)	58 (67.44)	49 (65.33)
Erythrocyte response					
Analyzed, n	68	169	153	68	59
Achieved, n (%)	55 (80.88)	132 (78.11)	122 (79.74)	55 (80.88)	48 (81.36)
Neutrophil response					
Analyzed, n	41	94	78	41	34
Achieved, n (%)	27 (65.85)	59 (62.77)	49 (62.82)	27 (65.85)	22 (64.71)
Trilineage response					
Analyzed, n	34	83	66	34	27
Achieved, n (%)	17 (50.00)	37 (44.58)	29 (43.94)	17 (50.00)	12 (44.44)

*AA* aplastic anemia, *ATG* anti-thymocyte globulin

**Fig. 5:**
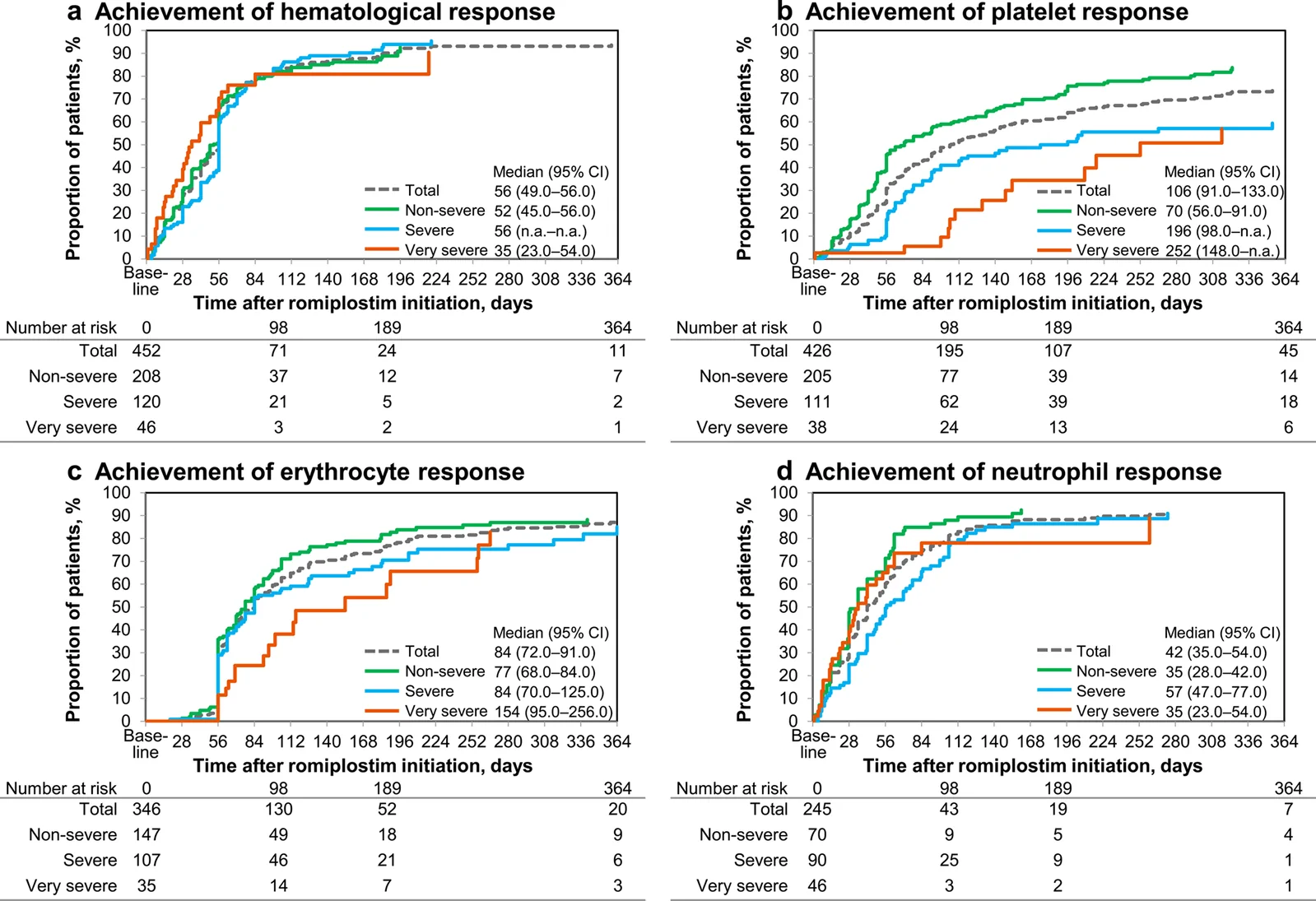
Kaplan-Meier plots for time to achievement of hematological response. Time to achievement of (**a**) hematological response, (**b**) platelet response, (**c**) erythrocyte response, and (**d**) neutrophil response. *CI* confidence interval, *n.a* not available

Using the Kaplan-Meier method (Fig. 5a–d), the median time (95% CI) to hematological response was estimated as 56 days (49.00, 56.00). Median times to lineage-specific responses were 42 days (35.00, 54.00) for neutrophils, 84 days (72.00, 91.00) for erythrocytes, and 106 days (91.00, 133.00) for platelets. Patients with very severe AA required longer times to achieve platelet and erythrocyte responses (Fig. 5b, c), whereas no clear differences were observed for hematological or neutrophil responses across severity groups (Fig. 5a, d).

**Fig. 6:**
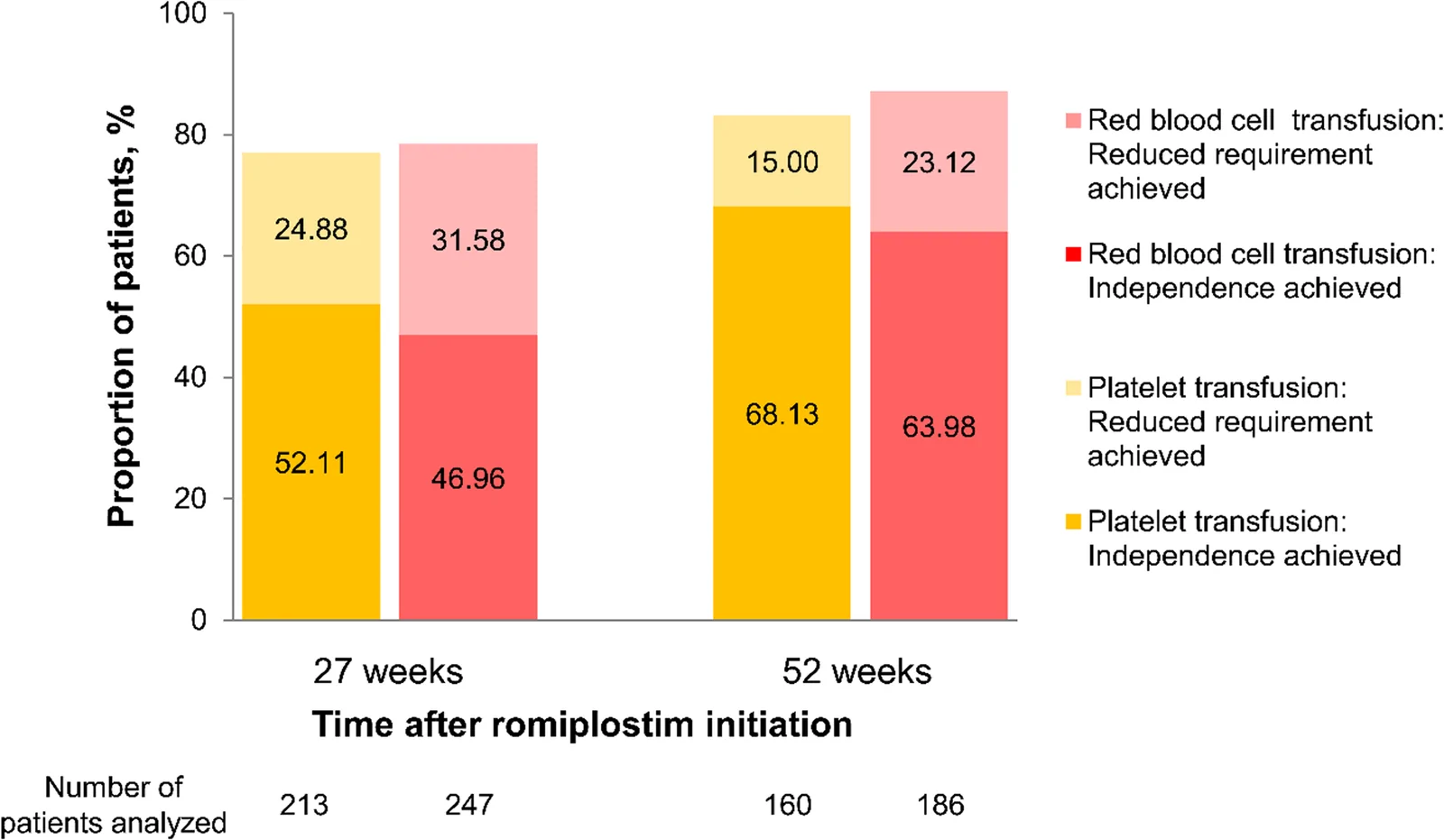
Platelet and red blood cell transfusion responses at 27 and 52 weeks after romiplostim initiation

**Fig. 7:**
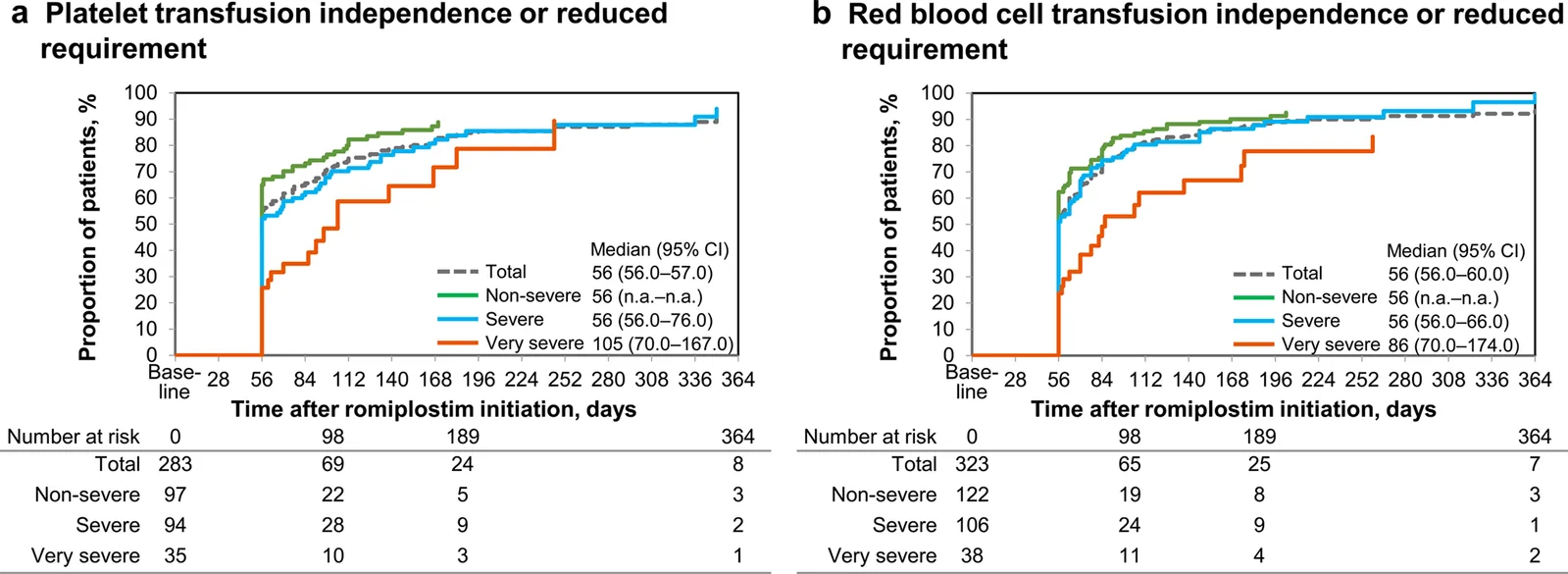
Kaplan-Meier plots for time to achievement of transfusion independence or reduced transfusion requirement. Time to achievement of transfusion independence or reduced requirement for (**a**) platelet transfusion and (**b**) red blood cell transfusion. *CI* confidence interval, *n.a* not available

After romiplostim initiation, platelet transfusion independence or reduced requirement was achieved in 164/213 patients (77.00%) at 27 weeks and in 133/160 patients (83.13%) at 52 weeks, with platelet transfusion independence achieved in 111/213 patients (52.11%) at 27 weeks and 109/160 patients (68.13%) at 52 weeks (Table 9). Similarly, RBC transfusion independence or reduced requirement was achieved in 194/247 patients (78.54%) at 27 weeks and in 162/186 patients (87.10%) at 52 weeks, with RBC transfusion independence achieved in 116/247 patients (46.96%) and 119/186 (63.98%) (Table 9). The proportion of patients who achieved either a platelet or RBC transfusion response increased over time (Fig. 6). The median time to transfusion response, estimated using the Kaplan-Meier method (Fig. 7), was 56 days for both platelet and RBC transfusion independence or reduced requirement, corresponding to the earliest evaluation time point defined by the study criteria (Table 1).

**Table 9:** Status of platelet and red blood cell transfusions at 27 and 52 weeks after romiplostim initiation

	Platelet transfusion		Red blood cell transfusion	
	27 weeks	52 weeks	27 weeks	52 weeks
Analyzed, n	213	160	247	186
Achieved, n (%)				
Transfusion independence or reduced requirement	164 (77.00)	133 (83.13)	194 (78.54)	162 (87.10)
Transfusion independence	111 (52.11)	109 (68.13)	116 (46.96)	119 (63.98)

## Discussion

This post-marketing surveillance study evaluated the safety and effectiveness of romiplostim in patients with refractory AA in a real-world setting. Compared with a phase II/III clinical study for romiplostim in patients with refractory AA conducted in Japan and Korea [29], patients in the present study were older (median age, 71 vs. 46 years) and had a shorter time since diagnosis (median, 1.1 vs. 4.5 years), while AA severity was similar (severe, 25.96% and very severe, 10.21% vs. severe, 29.0% and very severe, 12.9%), although baseline AA severity was evaluated at different time points: at diagnosis of AA in the clinical study [29] and at romiplostim initiation in the present study. Median platelet counts, hemoglobin concentrations, and neutrophil counts were also comparable between studies (1.30 × 10^4^/µL, 7.60 g/dL, and 911.24/µL in this study vs. 1.40 × 10^4^/µL, 7.1 g/dL, and 867/µL in the clinical study, respectively), indicating that the patient populations were not substantially different.

Romiplostim was discontinued in 219/470 patients (46.60%), most commonly because of lack of effectiveness (89/470 patients, 18.94%). Notably, in more than 25% of these cases (23/89 patients), treatment was discontinued at relatively low doses of romiplostim (≤ 15 µg/kg), suggesting that insufficient dosing may have contributed to perceived lack of response. In the absence of safety concerns, dose escalation may therefore be considered. ADRs occurred in 18.09% of patients in this study, compared with 9% in a phase II study of 35 Korean patients receiving 1–10 µg/kg [28] and 55% of patients in a phase II/III study of 31 Korean and Japanese patients [29]. As in clinical studies, individual ADRs were infrequent, and no specific ADR pattern was identified.

Liver disorder as an ADR was uncommon (2.34%), and treatment discontinuation because of liver disorder occurred in only 0.64% of patients. A meta-analysis showed a significantly higher incidence of liver dysfunction with eltrombopag combined with IST than with IST alone in patients with AA [33]. By contrast, the low incidence of liver disorder was observed with romiplostim in the present study suggests a relatively low risk of liver disorder by romiplostim during long-term treatment.

Four AEs (hemorrhage, thromboembolism, bone marrow fibrosis, and hematopoietic tumor) were predefined as AEs of special interest were determined to be AEs of special interest during the development phase of romiplostim for treatment of ITP. Activation of megakaryocytes via TPO receptor binding can induce megakaryocyte proliferation and bone marrow reticulin formation [34]. Accordingly, romiplostim, also a TPO-RA, could possibly induce reticulin formation in the bone marrow. In global studies of patients with ITP treated with romiplostim, reticulin formation in bone marrow was reported but was infrequent and generally transient [35, 36]. In clinical studies of patients with AA, neither reticulin formation nor its progression was observed [28, 29]. Consistent with these findings, no cases of bone marrow fibrosis were reported during the 2-year follow-up period in the present study. Although further monitoring is warranted, the risk of reticulin formation in bone marrow in patients with AA appears to be low.

In this study, hematopoietic tumors were observed in 11/470 patients (2.34%) during the observation period and in 5/310 patients (1.61%) during follow-up. While the incidence did not increase with longer exposure, the follow-up period was limited to 2 years, and long-term safety cannot be definitively assessed; therefore, caution is required for long-term administration. No hematopoietic tumors were reported in previous clinical studies involving Korean and Japanese patients [28, 29], and these studies enrolled fewer patients (66 in total) under strict inclusion criteria. The broader inclusion and larger sample size of the present real-world study may explain the detection of hematopoietic tumors. Importantly, AA itself has been known to associate with an increased risk of MDS and AML, with reported incidence rates of 4%–8% at 5–6 years and 9%–26% at 10 years in patients with AA treated with regimens other than TPO-RAs [37]. The incidence rate in the present study was not notably high relative to these previous reports, although patients were followed only for 2 years in our study.

Hematological response rates were 78.59% at 27 weeks and 84.02% at 52 weeks in our study, which were comparable to those reported in the phase II/III clinical study (83.9% and 80.6%, respectively) [29]. Trilineage response rates were also similar (37.68% at 27 weeks and 44.94% at 52 weeks in this study vs. 25.8% and 38.7%, respectively, in the clinical study) [29]. Furthermore, the rates for achievement of platelet transfusion independence or reduced requirement in the current study were also similar to those in the clinical study (77.00% at 27 weeks, 83.13% at 52 weeks in this study vs. 80.0% at both weeks in the clinical study) [29]. These results suggest that effectiveness in real-world practice is consistent with the efficacy observed in clinical trials.

At 52 weeks, hematological response rates were high across AA severity categories (84.62% in non-severe, 78.33% in severe, and 84.62% in very severe AA). Direct comparisons with eltrombopag, another TPO-RA, are not feasible because of differences in study populations, evaluation time points, and response criteria [38]. Nonetheless, a key finding of this study is that patients who were refractory to eltrombopag showed high hematological response rates to romiplostim (77.29% at 27 weeks and 81.50% at 52 weeks). Consistent with previous retrospective reports [30, 39], our findings support romiplostim as an effective alternative in this patient population.

This observational post-marketing study lacked a comparator arm, and differences in study design, eligibility criteria, and dosing preclude comparisons with clinical trials. Nevertheless, our results showing the effectiveness and safety of romiplostim in real-world practice is clinically meaningful for patients with AA, a rare disease with limited treatment options.

In conclusion, this all-case, non-interventional, prospective, observational post-marketing surveillance study conducted in Japan demonstrated that romiplostim is safe and effective in patients with refractory AA in a real-world setting.

## Data Availability

The anonymized data obtained and analyzed in this study are available upon reasonable request from a principal investigator.
